# Efficacy of Behavioral Experiments in Cognitive Therapy for Social Anxiety Disorder: Study protocol for a randomized controlled trial

**DOI:** 10.1186/s13063-019-3905-3

**Published:** 2019-12-19

**Authors:** Celina Clément, Jihong Lin, Ulrich Stangier

**Affiliations:** Department of Psychology, Clinical Psychology and Psychotherapy, Goethe University of Frankfurt, Varrentrappstraße 40-42, 60486 Frankfurt, Germany

**Keywords:** Social anxiety disorder, Social phobia, Cognitive behavioral therapy, Behavioral experiment, Treatment, Outcome

## Abstract

**Background:**

While the efficacy of cognitive therapy (CT) has been well established for social anxiety disorder (SAD) in several randomized controlled trials, there are still large differences between trials with respect to effect sizes. The present study investigates the question of whether enhanced training and the use of behavioral experiments (BEs) increases the efficacy of traditional CT, based on verbal methods of cognitive restructuring.

**Methods/design:**

A mixed within/between conditions design will be applied, with therapists and patients being randomly allocated to one of two conditions: (1) training of CT plus BEs, (2) training of CT “as usual”.

Sixty patients with the primary diagnosis of SAD will be recruited and treated in the outpatient clinic of the Department of Psychology, University of Frankfurt. To ensure adherence to therapist protocols, all therapists will be trained and supervised by the project coordinators. In addition, videotaped treatment sessions will be independently evaluated to guarantee both adherence to protocols and the quality of the intervention. Treatment effects will be assessed by independent SAD symptom ratings using the Liebowitz Social Anxiety Scale as the primary outcome measure and self-report measures as secondary outcome measures.

**Discussion:**

The present cognitive behavioral therapy (CBT) trial will be the first to clarify the contribution of BEs to the efficacy of CT in a randomized controlled design. Study results are relevant to clinical training and implementation of evidence-based treatments.

**Trial registration:**

German Clinical Trials Register International Clinical Trials Registry Platform (ICTRP) identifier: DRKS00014349. Trial status: recruiting.

## Background

Social anxiety disorder (SAD) is a highly prevalent and chronic psychiatric disorder associated with considerable psychosocial impairment. In recent years, growing evidence has suggested that individual cognitive therapy (CT) based upon the Clark and Wells model [1] may be superior to some alternative cognitive behavioral therapy (CBT) approaches, as well as to other treatment modalities. Six randomized controlled trials in three different countries have compared individual CT with alternative active treatments; individual CT was proven to be significantly more effective. Individual CT outperformed two versions of group CBT [2, 3], in vivo exposure [4], interpersonal psychotherapy [5], psychodynamic short-term psychotherapy [6], fluoxetine plus self-exposure instructions [4], and medication-based treatment as usual [3]. Mayo-Wilson et al. [7] reported a network meta-analysis of 101 randomized controlled trials on 41 psychological and pharmacological treatments and again demonstrated the highest effect sizes for Clark & Wells’ individual CT and a related individual CBT developed by Heimberg and colleagues.

Although the finding that individual CT is superior to other comparable treatments is consistent across studies in three European countries, Mayo-Wilson et al. [7] found that there was a significant difference in the magnitude of within CT change between English trials [4] [8] and German [2, 5, 6] and Swedish [3] trials. The standard mean difference compared to no treatment was − 1.56 for the English trials and − 0.97 for the German and Swedish trials.

There are several possible factors that may account for the differences between the two sets of trials. First, when comparing trials against the Clark et al. ones, the allegiance to the treatment may differ between researchers and therapists. Although the influence of researcher allegiance was minimized by the inclusion of D.M. Clark in the training, implementation and publication of the data of all trials, there are no data that allow for a direct comparison of therapist allegiance. Second, in the English trials almost all sessions were 90 min long to arrange the implementation of out of office behavioral experiments; however, in the German and Swedish trials, most therapists used shorter sessions lasting 50 min, which is in line with healthcare standards, but provides insufficient time to effectively set-up and discuss the results of in-session behavioral experiments. Evidence in support of this concern comes from a recent analysis [9] of video tapes and therapist protocols of CT treatments from the SOPHONET trial, a large-scale multicenter randomized controlled trial [6] comparing the efficacy of CT and short-term psychodynamic psychotherapy in Germany. Therapists protocols from *N* = 165 completed treatments disclosed which treatment interventions were actually applied in the sessions. Results indicated that almost all therapists applied basic cognitive interventions, such as deriving a cognitive model, roleplaying to avoid safety behaviors and self-attention, video feedback, and verbal techniques of cognitive restructuring (e.g., Socratic dialogue). In contrast, the frequency of behavioral experiments (BE) was significantly reduced compared to the required standard in the manual (the mean frequency in treatment was 1.7, versus a mean recommended frequency of 6). Furthermore, the analysis of the duration of the sessions revealed that the vast majority of sessions (on average 17 out of 25) lasted only 50 min. This indicates that the preference for 50-min sessions, as is usual in healthcare for psychotherapy settings, may have been one of the reasons for the low rate of BEs in the study.

However, there is some evidence that BE is a major effective component of CT. For instance, [10] found evidence that BE involving safety behaviors, attention manipulation, and video feedback are more effective in the treatment of SAD than traditional exposure. Thus, a possibly major effective component of CT was not implemented in the German trials and this failure may account for the differences in the effect sizes. However, as of yet, there is no direct evidence showing that the systematic use of BEs has a significant impact on the outcomes achieved with CT.

Given the possibility that the large differences in the effect sizes found for CT based on the Clark & Wells model may account for different amounts of BEs, there is an additional need to clarify the reasons for the insufficient implementation of BEs. Two factors may be responsible for the lack of adherence with this component of CT:

1. Lack of specific competence:

There is evidence that higher levels of general therapist competence are associated with better outcomes [9, 11]. In addition, Stangier et al. [9] found that in the SOPHONET trial, the specific competence to conduct BEs was significantly correlated with outcome (*r* = 0.28). Thus, training and supervision might have been insufficient to deliver adequate skills to plan and implement BEs as a routine component of CT.

2. Lack of therapists’ allegiance:

Although in vivo exposure has been proven to be an effective therapy for anxiety disorders, the majority of behavioral therapists in healthcare do not apply this method [12, 13]. There is evidence that one reason for the underutilization of exposure is negative beliefs about its effects [14]. In the context of BEs, negative expectations of their outcomes may have prevented therapists from conducting interventions. Although generally the outcomes of BEs will differ from the extreme expectations of patients, and even though the processing of negative results can be restructured upon subsequent reflection, therapists might prefer interventions with a lower risk of failure.

To summarize, the lack of specific competence and allegiance with BEs may account for the insufficient implementation of BEs and the lower efficacy of CT in the trials conducted in Germany compared to the trials conducted by the Oxford/London group. Consequently, intense training, the supervision of specific competencies, enhancing allegiance with this approach by including self-practice/self-reflection [15] in its training and establishing an appropriate setting (prolonged sessions, in situ guidance) to implement BEs is expected to significantly improve Standard CT.

### Hypotheses

The present study aims to investigate whether the enhanced implementation of BEs will improve the efficacy of CT for SAD. This goal will be achieved by the following measures:

1. comprehensive training of the therapists’ competencies in conducting in-session BEs, including self-practice and self-reflection,
2. encouraging therapists to implement this intervention as the major component of the treatment in continuous supervision,
3. establishing double hour sessions to ensure an appropriate setting for BEs.

The experimental condition (CT plus enhanced BEs) will be compared to standard CT as the control condition. An active control condition has been chosen as comparator to control for specific effects of conventional components of cognitive therapy. In standard CT, therapists’ training, supervision, and settings are arranged with a focus on traditional cognitive restructuring techniques.

In a randomized controlled trial, the following major hypothesis will be tested:

*Hypothesis 1: As compared to standard CT, CT plus enhanced BEs will result in a significantly higher rate of responders at (a) post-treatment and (b) follow-up.*

The secondary objective is to identify differences in the process of therapy and their association with treatment outcomes. The process-related variables to be assessed include the following therapist variables: general and treatment-specific competencies, and adherence to and allegiance with the treatment manual. The patient variables and treatment outcomes are: changes in insight in SAD-related beliefs, cognitions, avoidance, and symptoms, as well as comorbid depressive symptoms.

*Hypothesis 2: In the CT plus enhanced BEs, as compared to standard CT, therapists will show higher levels of adherence related to BEs, and significantly higher levels of allegiance with the treatment approach.*

*Hypothesis 3: In CT plus enhanced BEs, as compared to standard CT, changes in SAD-related insights, cognitions, avoidance, and symptoms (a) from baseline to post-treatment, and (b) from baseline to follow-up will be significantly increased.*

An additional objective is to identify outcome predictors, including therapist and patients’ variables and the therapeutic relationship.

*Hypothesis 4:*

*The following variables are expected to predict positive treatment outcomes:*

*Therapist variables: competencies, adherence, and allegiance; therapist-rated therapeutic relationship*

*Patient variables: preferred learning style at pre-treatment, changes in insights in SAD-related beliefs, changes in SAD-related cognitions; patient-rated therapeutic relationship.*

## Methods/Design

### Design and Sample Size

**Fig. 1:**
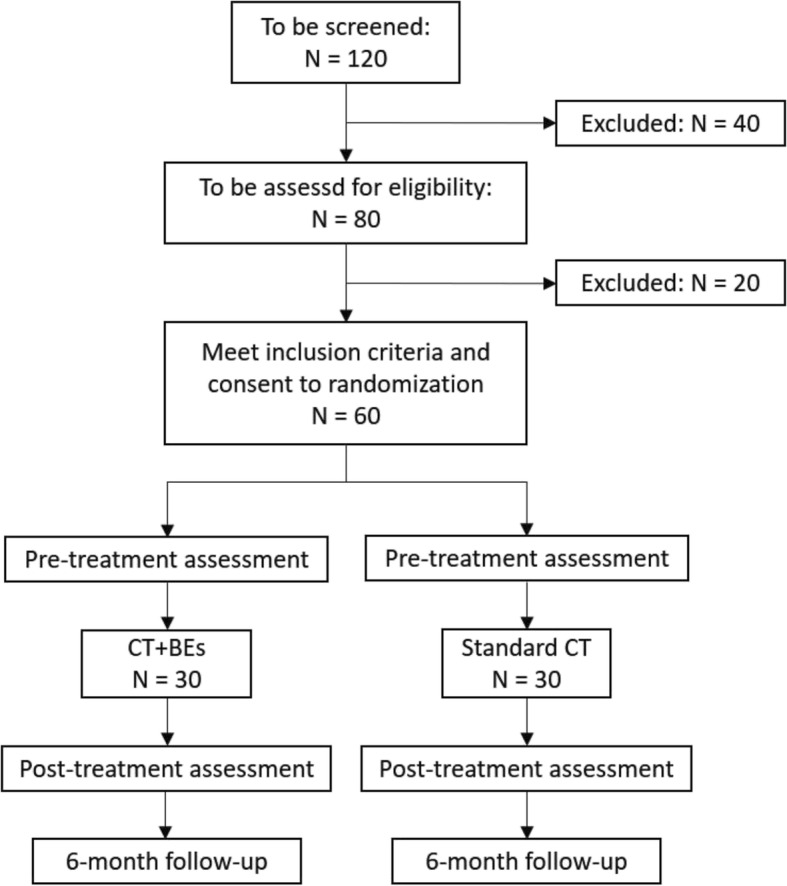
Study flowchart. Flowchart of planned participants’ selection and study design

The design of the study is a between group design with two treatment groups (CT plus enhanced BEs, standard CT). Patients will be randomly allocated to one of the two groups. The outcome criteria will be assessed by blinded independent raters at pre-treatment, post-treatment, and at follow-up 6months after post-treatment and treatment termination (Fig. 1).

The study is designed to detect a moderate effect size (d = 0.60, f = 0.35), as observed in the meta-analysis for CT for SAD with intense BE and prolonged sessions, as compared to CT with a reduced number of BEs and shortened sessions [7]. A power analysis with 90% power on a two-sided test where α = 0.05 revealed a sample size of *N* = 54 (27 per group). Based on data from previous studies [5], we expect a drop-out rate of 10%. Thus, 60 patients (30 per group) will have to be recruited.

### Study Procedures

All subjects contacted for the study will be screened over the telephone by trained master’s level research assistants. Those who seem eligible will be invited for two in-person assessments. The screening visit will include a diagnostic interview for Axis I and II disorders (SCID-I and SCID-II) and a semi-structured interview to assess the subject’s SAD and depression symptoms and insight into SAD-related beliefs (LSAS, SPIN, QIDS, BAPS). The diagnostic interviews will be conducted by trained [doctoral-level] independent raters who are blind to treatment conditions. Patients who are eligible will be randomized either to the CT plus enhanced BEs group or to the Standard CT group and invited for a baseline visit to complete a self-reported questionnaire. The outcome measures will be assessed by the independent raters upon the termination of treatment and after 6months. The study protocol adheres to the Standard Protocol Items: Recommendations for Interventional Trials (SPIRIT) checklist (Additional file 1).

### Recruitment

Patients will be recruited through the outpatient unit at Goethe University Frankfurt (Zentrum für Psychotherapie), and by mental health professionals, support groups, patient organizations, flyers at local sites (e.g., primary care), and advertisements in mass media and newspapers.

### Selection of Patients

Patients will be included if they meet the following criteria:

- primary diagnosis of SAD (German SKID modified for DSM-5)
- age 18–70 years
- no concurrent psychopharmacological or psychotherapeutic treatment
- written informed consent must be available before study participation

Patients presenting with any of the following criteria will not be included in the study:

- acute suicidality
- active substance abuse or dependence within the past 3 months
- severe depression
- psychotic disorder
- bipolar disorder
- borderline personality disorder
- organic mental disorder and/or severe medical conditions

Patients may be withdrawn from the study for the following reasons:

1. on request of the patient (withdrawal of the patient’s consent)
2. if continuation of the study would be detrimental to the subject’s well-being
3. occurrence of exclusion criteria

The investigator will decide whether to withdraw patients from the study in cases of 2) and 3). There are no specific plans to promote participant retention. Participants who discontinue will be asked to complete all assessments.

### Treatments

In the study, two conditions, CT plus enhanced BEs vs. standard CT, are compared; these use different interventions in the middle stage of treatment to achieve a change in SAD-related cognitions:

1. behavioral experiments
2. traditional (verbal) methods of cognitive restructuring.

CT based upon the Clark & Wells model pursues two primary goals:

1. to reverse the processes that maintain SAD (self-focused attention, distorted images of the social self, and safety behaviors), and
2. to disconfirm dysfunctional beliefs (using BE, imagery rescripting, and cognitive restructuring).

As described in detail in the revised manual [16], the treatment comprises the following interventions:

- Stage 1 (sessions 1–3): deriving an individual cognitive model, experiential exercises to avoid self-focused attention and safety behaviors, providing video feedback, and enhancing attention training;
- Stage 2 (sessions 4–13): BEs (in-session and homework) to test dysfunctional beliefs, and imagery rescripting to modify maladaptive beliefs linked to recurrent negative imagery;
- Stage 3 (14–17): termination of treatment (blueprint of techniques helpful for relapse prevention) and booster session.

To implement BEs, prolonged sessions are required to prepare the experiment, including defining the expectations to be tested, outlining the behaviors to be enacted in the experiment, and choosing an appropriate situation outside the therapy room. Thus, 2 × 50 min will be scheduled as the standard duration of the session.

Furthermore, guided discovery and standard CT discussion techniques are used in stage 2 to support the reflection of BEs consequences. However, no automatic thoughts records are used, and verbal techniques of cognitive restructuring are embedded in the planning of BEs and the analysis of the results. In contrast to standard CT, therapists will use the Social Cognitions Questionnaire (SCQ) on a weekly basis to determine the focus of the therapy.

As in standard CT, neither exposure based on a rational habituation nor formal social skills training is used.

Standard CT comprises similar elements in the first and third stage of therapy; however, in the middle stage (stage 2), discussion techniques of challenging dysfunctional beliefs are used instead of BE. The techniques are described in detail in the previous manual [17] and include Socratic dialogue, the downward arrow technique, the prejudice technique, the life chart technique, positive data log, automatic thoughts protocol, and the pie chart technique. Dysfunctional beliefs associated with early experiences are identified and discussed. Homework mainly focuses on automatic thoughts and the practice of Socratic dialogue.

In standard CT, therapists are not encouraged to use BEs. Neither exposure based on a habituation rational nor formal social skills training is used.

To equalize therapists’ contact time and treatment dose, both treatments comprise the same amount of contact hours. In line with short-time-therapy in Germany, 25 units of 50-min duration are provided. In CT + BEs, up to eight sessions last 100 min due to the implementation of BEs. Standard CT includes one session of 100-min duration. Thus, the total number of sessions differs (17 vs. 25). The length of both treatments, however, is 6 months, since booster sessions are conducted (4-week interval) in the end of CT + BEs as prescribed in the manual.

### Therapists

Therapists are recruited from courses in advanced clinical training for behavioral therapy and are randomly allocated to one of the two conditions. The randomization to either CT plus enhanced BEs or standard CT is uniquely conducted to prevent contamination.

In each condition, training comprises three 8-h days:

In the CT plus enhanced BEs condition, training will focus on the specific competencies to derive, plan, conduct, and analyze BEs.

In the standard CT condition, training will focus on the specific competencies to practice techniques of cognitive restructuring.

### Adherence Checks

Adherence refers to (a) the use of prescribed and avoidance of proscribed interventions by the manual, and (b) the extent to which the interventions are conducted in line with the manual. Thus, adherence will be measured in two ways:

1. After each session, therapists will report which interventions they administered using a checklist of interventions.
2. In addition, three randomly selected videotapes of each treatment (one from stage 1 and two from stage 2) will be rated on the Cognitive Therapy Adherence Scale (CTAS) [18]. Videotapes will be rated by qualified assessors who have been trained in previous projects and are experienced in the assessment of adherence to SAD-specific CT.

### Randomization

We will use two levels of randomization. Firstly, therapists are randomized to either CT + BEs or Standard CT as described above. Secondly, participants will be randomly assigned to a therapist of one of the two treatment conditions. The random allocation sequence will be generated by a randomization program [19]. Allocation to treatment of the patients is concealed from investigators and patients. The random allocation schedule will be drawn up and administered by a psychologist from another department of the university who is not involved in the further study process. After meeting the inclusion criteria, the participant is allocated by this psychologist to a therapist of one of the treatment conditions. The investigators and therapists are informed about the allocation.

### Ethical Issues

A protocol of the proposed project, informed consent document, and any other appropriate documents have been submitted to the independent Ethics Committee of Goethe University Frankfurt. Before the first subject is enrolled in the treatment study, all ethical and legal requirements have been met. The investigator will keep a record of all communications with the Ethics Committee and the regulatory authorities.

Before being admitted to the treatment study, the subject must consent to participate after the nature, scope, and possible consequences of the study have been explained in a form understandable to him or her. The subject must give written consent. A copy of the signed informed consent document will be given to the subject. The documents will be in a language understandable to the subject and will specify who informed the subject.

During the study, subjects will be identified solely by means of their initials, date of birth, and individual identification code (subject number, randomization number). Study findings stored on a computer will be stored in accordance with local data protection laws and will be handled in the strictest of confidence. Investigators will maintain a personal subject identification list (subject numbers with the corresponding subject names) to enable records to be identified.

Participating patients are provided with a treatment according to good clinical practice, i.e., CT according to the treatment guidelines of the German Psychological Association [20]. The cognitive interventions have no known risk, and no psychotherapy study for cognitive interventions in other psychiatric disorders has yet reported any adverse side effects. Suicidal tendencies will be checked regularly. If action is necessary, an outpatient service is available and admission to cooperating clinics is possible. The Center of psychotherapy, Goethe University Frankfurt will compensate to those who suffer harm from trial participation.

### Outcome Measures

The primary outcome measure is treatment response between baseline and posttreatment as assessed by the improvement item of the Clinical Global Impression Scale (CGI) modified for individuals with social anxiety disorder [21]. The CGI is a standard primary outcome measure in psychopharmacologic studies and has two components. The CGI-Severity (CGI-S) contains a Severity of Illness item that provides information about the current severity of social phobic symptoms. The severity rating is made by a clinician on a seven-step categorical scale with 1 representing “normal” or “not at all ill” and 7 representing “among the most severely ill patients”. The CGI-Improvement (CGI-I) rates the change in symptom severity and impairment from the baseline, ranging from 1 (“very much improved”) to 7 (“very much worse”). The psychometric properties of CGI have been found to be very good [5, 22]. The clinical raters assigned to each treatment condition will complete CGI-S at pre-treatment and CGI-I at post-treatment and 6-month follow-up assessment. Patients rated 1 or 2 (markedly or moderately improved from baseline) will be classified as responders, and those rated 3 or higher will be classified as non-responders.

The secondary outcome measures will be changes in the clinician-rated Liebowitz Social Anxiety Scale (LSAS) [23] and the patient-rated Social Phobia Inventory (SPIN) [24] from baseline to post-treatment. The LSAS assesses fear in and avoidance of 24 social situations in the last week, rated on 4-point scales ranging from 0 (non and never) to 4 (severe and usually) of. In the current study we will use the total score by summing both subscales. The SPIN consists of 17 items evaluating fear, avoidance, and physiological discomfort in the last week on a 5-point rating scale, ranging from 0 (not at all) to 4 (extremely). The total score is obtained by adding up the answers of the individual items.

Changes in insight in SAD-related beliefs from baseline to post-treatment and follow-up assessment will be assessed using the German version of the clinician-administered Brown Assessment of Beliefs Scale (BABS) [25–27]. The scale consists of seven items rating the degree of conviction and insight of beliefs from 0 to 4. The outcome measure will be the total score of the first six items.

In addition, symptoms of depression will be assessed by independent ratings conducted according to the German version of the Quick Inventory of Depressive Symptomatology (QIDS; 16-item version) [28] and the Beck Depression Inventory (BDI-II) [29]. For both inventories we will use total score to examine changes from baseline to post-treatment and to follow-up assessment.

Each outcome measure will be completed at the pre-treatment, at post-treatment and 6-month follow-up.

Clinical ratings are administered by independent raters blinded to condition. Raters will be trained to perform CGI, LSAS, QIDS, and BABS assessments. The interviews will be videotaped to review and assess inter-rater reliability between independent raters.

### Additional Assessments

For competence and adherence ratings, three videotapes will be selected: one from the initial stage (session 1, deriving the model) and two from the second stage (random selection from session 5–12). Session 1 is selected to cover general competencies such as guided discovery, whereas sessions from the second stage of treatment onwards are expected to refer to specific competencies (conducting BEs vs. cognitive restructuring). Competence and adherence will be rated by two independent, masked, and trained raters using the Cognitive Therapy Adherence Scale for Social Phobia (CTAS-SP) [18] the Cognitive Therapy Competence Scale for Social Phobia (CTCS-SP) [30]. The CTAS-SP comprises 17 items related to the specific interventions of CT, as described in the manual by Stangier et al. [17]. In the SOPHONET trial [6], a good interrater reliability (ICC = 0.87) was observed. Retest reliability was rtt = 0.90 for the total score and rtt = 0.28–1.0 for the individual items. Internal consistency was high (α = 0.99). Similarly, the psychometric properties of the 16-item CTCS-SP were good, with an interrater reliability of ICC = 0.81, retest reliability of rtt = 0.92, and an internal consistency of α = 0.97.

To assess the therapeutic relationship, the Helping Alliance Questionnaire (HAQ, patient and therapist version) will be used [31]. Both questionnaires will be administered at pre-treatment. The mean scores of the therapist and patient versions will be used to predict the outcomes.

Allegiance is assessed after the training and one pilot-therapy by the therapist version of the Reaction to Treatment Questionnaire developed by Holt & Heimberg [32]. For the purpose of the present study, two further items have been added to the scale referring to outcome expectations about specific intervention (BEs and cognitive restructuring).

As a predictor of treatment success, a 12-item version of the learning style inventory (Kolb LSI) will be administered at pre-treatment [33]. Based on previous treatment study results [34], it is hypothesized that the outcome of participants in the BE intervention will significantly predict an active experimentation learning style, whereas for participants in the standard CT (cognitive restructuring), the preferred learning style is predicted to be an abstract conceptualization learning style.

The SCQ (Social Cognition Questionnaire) [35] (German translation [36]) is a self-report measure to assess the frequency and intensity of SAD-related cognitions. The questionnaire will be completed by the patients at every session.

**Fig. 2:**
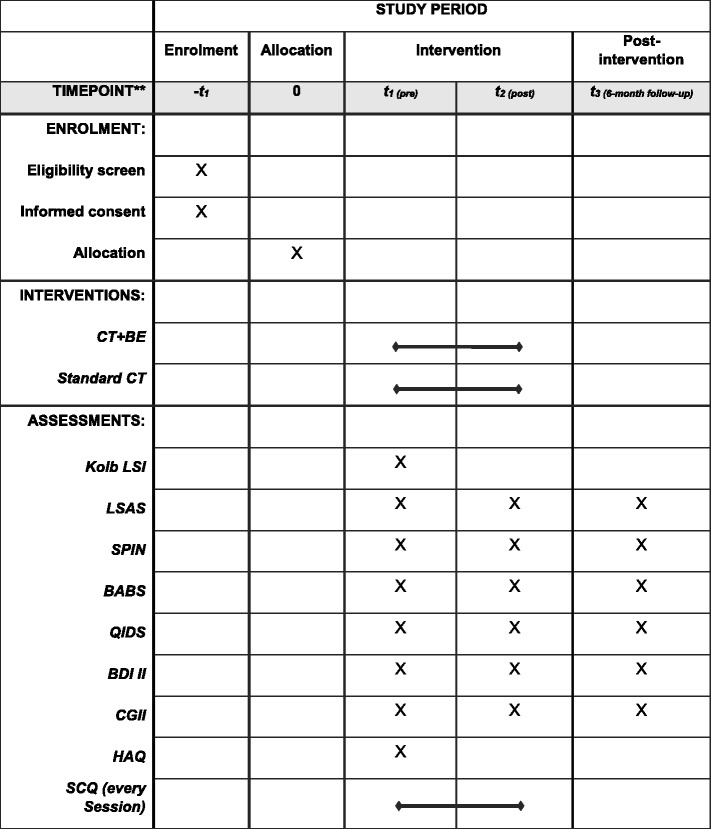
Schedule of enrolment, interventions, and assessments

Detailed procedure of the measurements is listed in the schedule of enrolment, interventions and assessments (Fig. 2).

## Statistical Procedures

### Analysis of Outcome Variables

The primary outcome measure of the study is the proportion of treatment responders at post-treatment rated by the CGI-I. We dichotomize the variable so that treatment responders are defined by a rating of 1 or 2 (markedly or moderately improved from baseline) and non-responders by a rating of 3 or higher [5, 22]. The secondary outcome variables are changes in the mean scores of LSAS and SPIN from baseline to post-treatment. In addition, the primary and secondary outcome variables will be analyzed at 6-month follow-up to examine changes from baseline to follow-up assessment.

### Statistical Analyses

Data will be analyzed with the SPSS statistical software package. All statistical analyses will be conducted on the intent-to-treat (ITT) sample and completer sample. Patients allocated to treatments will be classified as drop-outs if they miss more than 25% of the sessions. In the ITT analyses of treatment response, drop-outs will be considered as failures. In ITT analyses of dimensional measures, missing data will be replaced using the last observation carried forward approach for missing intervention- and post-assessments or follow-up observations. Analyses of the primary outcome measure (CGI-I), a categorical variable, will be conducted using χ^2^ or Fisher's exact tests. Dimensional measures will be submitted to analyses of covariance (ANCOVAs) in which pre-treatment scores will be controlled for. ANCOVAS will be performed separately for the post-treatment and 6-month follow-up assessments. Significance levels will be set at *p* < 0.05, two-tailed. Predictor analysis will be calculated by using logistic regression analysis with CGI-I as the dependent variable; independent variables are competencies, adherence, allegiance, and therapist-rated therapeutic relationships (therapist predictors), as well as patients’ preferred learning style at pre-treatment, changes in insight in SAD-related beliefs and cognitions, and patient-rated therapeutic relationships (patient predictors). Differences between treatments in the outcome predictions will be analyzed, with treatment interactions for each predictor. To test for possible therapist differences, a preliminary analysis of covariance will be conducted. As recommended, significance level in this case will be set at *p* < .30 [37]. If the effect is significant, a multilevel analysis using a mixed-model design is planned to include therapists as a random term [38]. A final analysis will be run to compare the pattern of results from both strategies of analysis taking possible therapist effects into consideration.

## Discussion

This study is the first to determine the efficacy of BE as a component of CT. Several previous studies have shown the overall efficacy of CT including BE [7], but the incremental efficacy of BE as an additive intervention has yet to be verified. BEs imply exposure to critical social situation, but not with the explicit goal of achieving habituation; rather, it is to test dysfunctional beliefs [39]. Thus, BEs go beyond traditional verbal methods of cognitive restructuring, including both Socratic dialogue and the automatic thoughts questionnaire, which involve the logical disputation of dysfunctional beliefs and aim to provide insight. BEs, however, by approaching critical and frequently avoided social situations, are suggested to provide information challenging dysfunctional beliefs. Thus, in contrast to traditional exposure, BEs explicitly refer to cognitive mechanisms; in particular, encoding new information into memory [40]. Although traditional exposure may implicitly involve similar mechanisms, BEs not only aim to change dysfunctional beliefs, but in addition target the modification of the cognitive processes that maintain them, i.e., self-focused attention, as well as safety behaviors and distorted images [41]. The results of a recent meta-analysis [10] indicate that BEs might be superior to exposure in some anxiety disorders, among them SAD; however, the results do not clarify whether BEs contribute significantly to the efficacy of CT in general. Furthermore, the superiority of the behavioral experiment condition in this review could possibly reflect therapeutic-allegiance effects since most of the research groups did have recognized allegiance to CT. Therapeutic allegiance, however, improves the outcome of treatment and may place treatment conditions with higher allegiance in a more favorable position compared to competing treatment conditions.

In the current study, the two active treatment conditions differ with respect to one component: BE. Thus, this study may be categorized as a dismantling study [42, 43], decomposing a multi component therapy by removing one component expected to be an effective ingredient. The expected effect size for the difference between a treatment including or not including one component usually is expected to be small, requiring large sample sizes to detect significant differences. For instance, a meta-analysis by Bell et al. [43] confirmed that the effects in dismantling studies were very small and nonsignificant. Thus, a high number of participants is needed to detect significant differences. However, Bell et al. also found that additive studies show significant, although small, effects of additive components. Thus, it is of importance whether an effective treatment package is decomposed, or an effective treatment component is added. The present study may rather be classified as an additive study, because a new component (BEs aiming at new emotional experiences) is added to traditional CT (using mainly verbal methods to achieve insight). In our study, BEs are a vital part in the therapy. Therapists are encouraged to implement BEs over a large number of sessions, so the differences between both groups might be greater than in conventional dismantling studies.

Another potential limitation that may impair the validity of the previous studies comparing BEs to exposure is the allegiance of researcher and therapists. Much of the effects of psychological treatments may be attributed to the effect of allegiance [44]. Therefore, we attempt to control for possible biases due to an imbalance between both conditions by several measures: Firstly, we randomize therapists to one of both conditions and train them separately. Secondly, although missing behavioral experiments, we prevent a possible devaluation of the control condition by emphasizing the particular benefit of cognitive restructuring for strengthening self-esteem. Thirdly, we will check for possible differences in allegiance between both conditions using the therapist version of the Reaction to Treatment Questionnaire developed by Holt & Heimberg [32].

In addition, to ensure equal allegiance, we also try to control for differences between conditions in adherence. Both groups of therapists receive detailed manuals [16], extended training, and continuous supervision. An additional adherence check will be established by the analysis of videotaped sessions [11].

A focus of the training in the experimental conditions is to overcome barriers, that have also been found in the implementation of exposure into practice. One of the reasons for the underutilization of in vivo exposure in previous studies could be a lack of flexibility in terms of time and location, which is necessary to approach the anxiety provoking situation and implement exposure and response prevention until habituation [45]. Similar to in vivo exposure [46], the presence of the therapist in situ is required to overcome avoidance, identify and drop safety behaviors, ensure that the critical behavior is performed, model the critical behavior to allow for observational experiments, instruct the patient to externalize attention and accurately assess other people’s reactions. However, the preference for 50-min sessions in the German health care setting may have limited the conditions to leave the therapy room and guide BEs in situ. Thus, to support flexibility we plan double sessions as obligatory part of CT + BEs. The contact time and use of prolonged sessions will be assessed by the therapist after every session.

In addition, specific competencies are needed to conduct BEs and build up motivation for approaching anxiety-provoking situations. Therefore, therapists receive detailed instructions, e.g., how to operationalize key beliefs into concrete expectations related to specific situations, how to encourage the patient to approach this situation, to instruct the patient to drop safety behaviors and self-attention, to encourage the patient to enact critical behavior related to feared outcomes (e.g., blushing), and to review the actual outcome in relationship to expected outcome. An additional part of the training will be a one pilot-therapy of the first patient to consolidate the competence and counteract negative expectations. For further support, we provide continuous supervision by the senior investigator.

The intense supervision was a reason why we decided to recruit all therapists from our affiliated center for training. Furthermore, therapists’ clinical experience will be comparable with CT therapists from the German SOPHONET trial (M = 1.7 years, SD = 0.9) [6]. In the trial, there was no association between therapists’ experience and remission or response rate. However, this has the potential to limit the generalizability of the trial results to routine clinical care. Besides, the intense supervision could be a further limitation of generalizability too.

To summarize, the results of this study will further the literature of the additive efficacy of a component that is expected to be a major effective ingredient of CT. In addition, it may explain parts of the variance in the outcome of different studies referring to the same treatment approach, but showed different levels of adherence.

Finally, it will contribute to the dissemination of BE as a treatment component, which is often neglected in psychotherapy research and healthcare.

### Organizational structure and responsibilities

**Trial Management Committee (senior investigator, co-investigators)**

Design and conduct of the study

Study planning

Interpretation of results

Publication of study reports

**Co-investigators**

Reviewing progress of study (completeness, accuracy, and timeliness of data collection)

**Graduate student research assistants**

Recruitment

Screening

**Senior investigator**

Training of therapists and independent clinical raters.

Supervision

Reviewing adherence to study protocol, reporting of harms

**Steering committee**

Due to the limited scale of this study no steering committee has been planned. The responsibilities of a steering committee will be taken on by the TMC.

**Data monitoring committee (DMC)**

There will be no DMC since we offer a short effective treatment in both conditions with known minimal risks. No interim analyses have been planned.

**Data monitoring and quality assurance**

There will be no auditing process by an institution or agency independent from the investigators. The integrity of data quality is ensured by independent clinical raters assessing inclusion criteria and primary outcome. Random allocation to treatment will be administered by a psychologist from another department of the university. Adherence to trial interventions is ensured by checklists of interventions endorsed by therapists after each session, as well as videotapes of session. Supervision of therapists is provided separately by either the senior investigator for the experimental condition, or by an independent supervisor for the control condition. Allegiance of therapists in both study conditions is assessed after the pilot therapy. Completeness, accuracy, and timeliness of data collection is checked by the Co-investigators. Serious adverse events and harms are reported to the senior investigator, who will conduct interviews and refer the patient to crisis intervention in the center for psychotherapy affiliated to the department of psychology.

## Supplementary information

**Additional file 1.** SPIRIT 2013 Checklist: Recommended items to address in a clinical trial protocol and related documents.

## Data Availability

The datasets used and analyzed during the current study are available from the corresponding author on reasonable request.

## Abbreviations

ANCOVA: Analyses of covariance
BABS: Brown Assessment of Beliefs Scale
BDI-II: Beck Depression Inventory
BE: Behavioral experiment
CBT: Cognitive behavioral therapy
CGI-I: Clinical Global Impression Improvement Scale
CGI-S: Clinical Global Impression Severity Scale
CT: Cognitive therapy
CTAS-SP: Cognitive Therapy Adherence Scale for Social Phobia
CTCS-SP: Cognitive Therapy Competence Scale for Social Phobia
HAQ: Helping Alliance Questionnaire
ICC: Interrater reliability
ITT: Intent-to-treat
LSAS: Liebowitz Social Anxiety Scale
QIDS: Quick Inventory of Depressive Symptomatology
rtt: Re-test reliability
SAD: Social anxiety disorder
SCID-I: Diagnostic interview for Axis I disorder
SCID-II D: Diagnostic interview for Axis II disorder
SCQ: Social Cognition Questionnaire
SPIN: Social Phobia Inventory
